# Evaluation of recurrence risk for patients with stage I invasive lung adenocarcinoma manifesting as solid nodules based on ^18^F-FDG PET/CT, imaging signs, and clinicopathological features

**DOI:** 10.1186/s13550-023-00998-z

**Published:** 2023-06-01

**Authors:** Xuan Zheng, Jie Lin, Jiageng Xie, Jia Jiang, Junping Lan, Xiaowei Ji, Kun Tang, Xiangwu Zheng, Jinjin Liu

**Affiliations:** 1 Department of Radiology, The First Affiliated Hospital of Wenzhou Medical University, Wenzhou, 325000 Zhejiang China; 2 Department of Nuclear Medicine, The First Affiliated Hospital of Wenzhou Medical University, Wenzhou, 325000 Zhejiang China

**Keywords:** Adenocarcinoma of lung, Positron emission computed tomography, Prognosis, Risk factors, Proportional hazards models

## Abstract

**Background:**

Stage I lung adenocarcinoma is a heterogeneous group. Previous studies have shown the prognostic evaluation value of PET/CT in this cohort; however, few studies focused on stage I invasive adenocarcinoma manifesting as solid nodules. This study aimed to evaluate the recurrence risk for patients with stage I invasive lung adenocarcinoma manifesting as solid nodules based on ^18^F-FDG PET/CT, CT imaging signs, and clinicopathological parameters.

**Methods:**

We retrospectively enrolled 230 patients who underwent ^18^F-FDG PET/CT examination between January 2013 and July 2019. Metabolic parameters: maximum standard uptake value (SUVmax), mean standard uptake value, tumor metabolic volume (MTV), and total tumor glucose digestion were collected. Kaplan–Meier method was used to evaluate recurrence-free survival (RFS), and the multivariate Cox proportional hazards model was used to determine the independent risk factors associated with RFS. The time-dependent receiver operating characteristic curve (ROC) method was used to calculate the optimal cutoff value of metabolic parameters.

**Results:**

The 5-year RFS rate for all patients was 71.7%. Multivariate Cox analysis revealed that the International Association for the Study of Lung Cancer Pathology Committee (IASLC) pathologic grade 3 [Hazard ratio (HR), 3.96; 95% Confidence interval (CI), 1.11–14.09], the presence of cavity sign (HR 5.38; 95% CI 2.23–12.96), SUVmax (HR 1.23; 95% CI 1.13–1.33), and MTV (HR 1.05; 95% CI 1.01–1.08) were potential independent prognostic factors for RFS. Patients with IASLC grade 3, the presence of cavity sign, SUVmax > 3.9, or MTV > 5.4 cm^3^ were classified as high risk, while others were classified as low risk. There was a significant difference in RFS between the high-risk and low-risk groups (HR 6.04; 95% CI 2.17–16.82, *P* < 0.001), and the 5-year RFS rate was 94.1% for the low-risk group and 61.3% for the high-risk group.

**Conclusions:**

We successfully evaluate the recurrence risk of patients with stage I invasive adenocarcinoma manifesting as solid nodules for the first time. The 5-year RFS rate in the high-risk group was significantly lower than in the low-risk group (61.3% vs. 94.1%). Our study may aid in optimizing therapeutic strategies and improving survival benefits for those patients.

## Introduction

Lung cancer remains the leading cause of cancer-related mortality worldwide [1, 2], of which non-small cell lung cancer (NSCLC) is the most common type. The recommended treatment for the operable NSCLC is lobectomy with systematic lymph node dissection; the tumor node metastasis (TNM) staging system is a valuable prognostic tool and guides treatment decisions for NSCLC [3, 4]. However, patients with stage I NSCLC may still develop recurrence [5]. 18–32% of these patients died within five years after surgery, most of them due to recurrent, and the 5-year recurrence-free survival (RFS) rates were 84.3% for stage IA and 65.8% for stage IB [3]. Therefore, stage I NSCLC is a heterogeneous group. TNM staging alone may not be sufficient to predict the recurrence risk of early-stage NSCLC, and additional prognostic biomarkers are required.

With the development of high-resolution CT and the widespread use of low-dose CT screening for tumor screening, early-stage lung cancer has frequently been detected, especially small-sized lung adenocarcinoma. Lung adenocarcinoma is the primary subtype of NSCLC, accounting for approximately 70% of all NSCLC cases. Solid nodules are associated with a higher risk of recurrence than nonsolid and part-solid types [6]. Screening patients at high risk of recurrence is significant since further individualized treatment, such as adjuvant chemotherapy, might improve survival in those patients [7]. For solid nodules ≥ 8 mm on initial screening low-dose CT, PET/CT is recommended for lung cancer screening [8]. Previous studies [9–11] have confirmed the prognostic value of PET/CT parameters for NSCLC patients, such as the maximal standardized uptake value (SUVmax), metabolic tumor volume (MTV), and total lesion glycolysis (TLG), and further calculated the optimal cutoff values of these PET/CT parameters. However, different optimal cutoff values of these metabolic parameters have been reported. To our knowledge, the prognostic value of PET/CT combined with imaging signs and clinicopathological parameters to stratify patients at a high risk of recurrence for stage I invasive lung adenocarcinoma manifesting as solid nodules of the lung has rarely been reported.

This study aimed to evaluate the postoperative recurrence risk for patients with solid lung nodules in stage I invasive adenocarcinoma based on ^18^F-FDG PET/CT, CT imaging signs, and clinicopathological parameters. These patients at high risk of recurrence would be candidates for adjuvant therapy and tailored surveillance regimens.

## Materials and methods

### Patients

Our institutional review board approved this study, waiving the written informed consent requirement. We retrospectively reviewed medical records from the PACS system to identify patients with pathologic stage I (i.e., T1-2aN0M0) invasive nonmucinous adenocarcinoma manifesting as solid lung nodules between January 2013 and July 2019. All the included patients underwent ^18^F-FDG PET/CT scans within one month before surgical resection and had complete follow-up records. We excluded patients diagnosed with other malignant tumors and those treated with neoadjuvant chemotherapy or radiation before surgery. Patients with missing preoperative images or survival data were also excluded from this study.

We collected patients’ demographic, clinicopathological, and recurrence data. CT imaging signs included lobulation, speculation, cavity, air bronchogram, vessel convergence, and pleural indentation. The cavity sign represents air space within a primary tumor, excluding tumors with traction bronchiectasis and lung cysts [12]. Two experienced radiologists, blinded to the patient’s diagnosis and treatment information, independently evaluated these CT imaging signs; the discrepancy between them was resolved by discussion until reaching a consensus. PET metabolic parameters included SUVmax, MTV, TLG, and the mean standardized uptake value (SUVmean). The endpoint of this study was RFS [13], which was defined as the period from surgical resection to the occurrence of recurrence or death from any cause. RFS was censored at the last visit for those alive and recurrence-free. Recurrence was defined by where it occurs, including local, regional, and distant recurrence.

### Pathologic diagnosis and grading criteria

Pathologic TNM staging was determined according to the American Joint Committee on Cancer TNM Staging Manual, 8th Edition [14]. Among stage I tumors, the following tumor-size groups were created: T1a, ≤ 1 cm; T1b, > 1 to 2 cm; T1c, > 2 to 3 cm; and T2a, > 3 to 4 cm. Pathologic grading was evaluated using the grading system for invasive pulmonary adenocarcinoma based on the predominant histologic plus high-grade patterns from the International Association for the Study of Lung Cancer Pathology Committee (IASLC) [15]. The new detailed definition of IASLC grading was as follows: grade 1, well-differentiated, lepidic dominant with no or less than 20% of high-grade patterns; grade 2, moderately differentiated, acinar or papillary predominant with no or less than 20% of high-grade patterns; and grade 3, poorly differentiated, any tumor with 20% or more high-grade patterns. High-grade patterns included solid, micropapillary, and complex glandular patterns.

### FDG PET/CT imaging

The FDG PET/CT imaging protocol followed the EANM procedure guidelines [16]. All patients were required to have a fasting period of at least 6 h and a blood glucose level below 110 mg/dl before administration of ^18^F-FDG. PET/CT images were acquired 60 min after intravenous injection of 3.7 MBq/kg dose of ^18^F-FDG using a hybrid PET/CT scanner (GEMINI TF 64, Philips, Netherlands). A nonenhanced CT scan was performed from the skull base to the middle of the thigh for precise anatomical location and attenuation correction, and the corresponding scanning parameters were as follows: 120 kVp, 80 mAs, pitch of 0.829, tube rotation time of 0.5 s per rotation, and both slice thickness and interval of 5.0 mm. A 3-dimensional mode PET scan, which matched the CT slice thickness, was then performed. PET datasets were iteratively reconstructed using the ordered subset expectation maximization algorithm.

Two experienced nuclear medicine physicians analyzed all PET, CT, and PET/CT fusion images using Philips Extend Brilliance Workstation 3.0. SUVmax was determined as the highest voxel value within the VOI. We delineated the volume of interest (i.e., tumor region) using the SUV threshold method. MTV represents the sum of voxels with SUV no less than 40% of the SUVmax. SUVmean represented the mean SUV in the VOI. TLG was the product of MTV and SUVmean.

### Statistical analysis

We performed statistical analyses using the software SPSS (version 22.0; IBM Corp., Armonk, New York, USA). Continuous variables were presented as the mean value ± standard deviation, and categorical variables as the frequency (percentage). Kaplan–Meier method was used to determine RFS, and a log-rank test was applied to evaluate the difference of RFS between groups. The univariable Cox proportional hazard model was used to examine the significance of the clinicopathological features and imaging findings as predictors of RFS. Variables with *P* < 0.05 were used in the multivariable Cox proportional hazard model with the stepwise selection method, which was used to identify independent prognostic factors of RFS. The time-dependent receiver operating characteristic curve (ROC) [17] was used to estimate the optimal cutoff values of continuous predictors by using the software package survivalROC in the R programming environment (version 3.6.1). Statistical significance was set at *P* < 0.05.

## Results

**Fig. 1:**
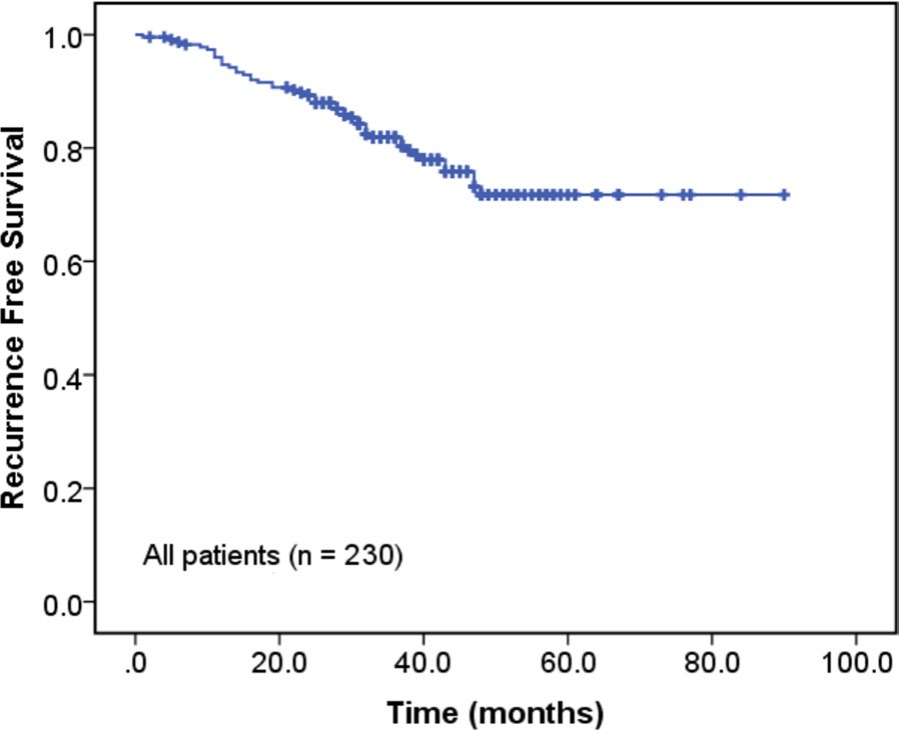
Kaplan–Meier analysis for all patients

We enrolled 230 patients in this study. Table 1 illustrates the baseline characteristics of these patients. The median follow-up time after surgical resection was 35.5 months. The mean age was 62.7 ± 9.7 years old, and 111 (48.3%) were men. The mean tumor size was 20.9 ± 8.0 mm. The number of patients was 19 (8.3%) in stage T1a, 111 (48.3%) in T1b, 66 (28.7%) in T1c, and 34 (14.8%) in T2a. There were 29 (12.6%), 177 (77.0%), and 24 (10.4%) patients with proposed IASLC grades 1, 2, and 3, respectively. Figure 1 shows the Kaplan–Meier analysis for all patients. The 5-year RFS rate for all patients was 71.7%.

**Table 1:** Patient characteristics (*n* = 230)

Variables	Value
Age (year)	62.7 ± 9.7
Male	111 (48.3%)
Smoking (yes)	69 (30.0%)
Hypertension (yes)	87 (37.8%)
Diabetes mellitus (yes)	27 (11.7%)
*Lesion location*	
Right upper lobe	66 (28.7%)
Right middle lobe	27 (7.4%)
Right lower lobe	52 (22.6%)
Left upper lobe	59 (25.7%)
Left lower lobe	36 (15.7%)
*T stage*	
T1a	19 (8.3%)
T1b	111 (48.3%)
T1c	66 (28.7%)
T2a	34 (14.8%)
*IASLC grading*	
Grade 1	29 (12.6%)
Grade 2	177 (77.0%)
Grade 3	24 (10.4%)
*Imaging sign*	
Lobulation (yes)	95 (41.3%)
Spiculation (yes)	55 (23.9%)
Cavity (yes)	8 (3.5%)
Air bronchogram (yes)	22 (9.6%)
Vessel convergence (yes)	10 (4.3%)
Pleural indentation (yes)	90 (39.1%)
Tumor size (mm)	20.9 ± 8.0
CEA (μg/L)	4.6 ± 7.0
CYFRA21-1 (ng/ml)	2.6 ± 1.3
SCCA (μg/L)	0.9 ± 0.6
NSE (ng/ml)	12.8 ± 3.4
ProGRP (ng/L)	49.6 ± 25.0
Albumin (g/L)	40.3 ± 4.4
White blood cell (X10^9^/L)	5.78 ± 1.73
Neutrophil-to-lymphocyte ratio	2.06 ± 1.10
Platelet-to-lymphocyte ratio	0.27 ± 0.11
SUVmax	4.7 ± 3.0
SUVmean	2.8 ± 1.8
MTV (cm^3^)	6.2 ± 5.9
TLG	18.7 ± 24.9

*IASLC* International Association for the Study of Lung Cancer Pathology Committee, *CEA* Carcinoembryonic antigen, *CYFRA21-1* fragment of cytokeratin 19, *NSE* Neuron Specific Enolase, *SCCA* squamous cell carcinoma antigen, *ProGRP* Progastrin-releasing peptide, *SUVmax* maximum standard uptake value, *SUVmean* mean standard uptake value, *MTV* metabolic tumor volume, *TLG* total lesion glycolysis

Table 2 shows the univariable and multivariable Cox analyses for 5-year RFS. In univariate analyses, IASLC grading, tumor size, T stage, the presence of cavity sign, SUVmax, SUVmean, MTV, and TLG were significant predictors of RFS. In multivariate Cox analyses, IASLC grade 3 (hazard ratio [HR], 3.96; 95% confidence interval [CI], 1.11–14.09), the presence of cavity (HR 5.38; 95% CI 2.23–12.96), SUVmax (HR 1.23; 95% CI 1.13–1.33), and MTV (HR 1.05; 95% CI 1.01–1.08) were identified as independent prognostic factors for RFS.

**Table 2:** Univariate and multivariate analysis for recurrence-free survival

Variable	Univariable analysis			Multivariable analysis		
	HR	95% CI	*P*	HR	95% CI	*P*
Age (years)	0.997	0.968–1.028	0.861			
Male	1.051	0.595–1.857	0.863			
Smoking						
Hypertension	1.468	0.831–2.593	0.186			
Diabetes	1.237	0.555–2.758	0.603			
IASLC grading			< 0.001			< 0.001
Grade 1 (Ref.)						
Grade 2	1.556	0.547–4.427	0.407	1.265	0.393–4.077	0.694
Grade 3	7.083	2.321–21.655	0.001	3.957	1.111–14.092	0.034
Tumor size	1.082	1.046–1.120	< 0.001			
T stage	2.221	1.589–3.102	< 0.001			
Lobulation	1.274	0.723–2.246	0.402			
Spiculation	0.813	0.405–1.632	0.560			
Cavity sign	4.912	2.087–11.563	< 0.001	5.381	2.234–12.961	< 0.001
Air bronchogram	0.609	0.189–1.961	0.406			
Vessel convergence	0.424	0.058–3.072	0.395			
Pleural indentation	1.173	0.663–2.075	0.584			
SUVmax	1.247	1.163–1.336	< 0.001	1.225	1.133–1.326	< 0.001
SUVmean	1.412	1.266–1.574	< 0.001			
MTV	1.037	1.010–1.065	0.006	1.045	1.009–1.082	0.045
TLG	1.018	1.011–1.024	< 0.001			

*SUVmax* maximum standard uptake value, *SUVmean* mean standard uptake value, *MTV* metabolic tumor volume, *TLG* total lesion glycolysis

**Fig. 2:**
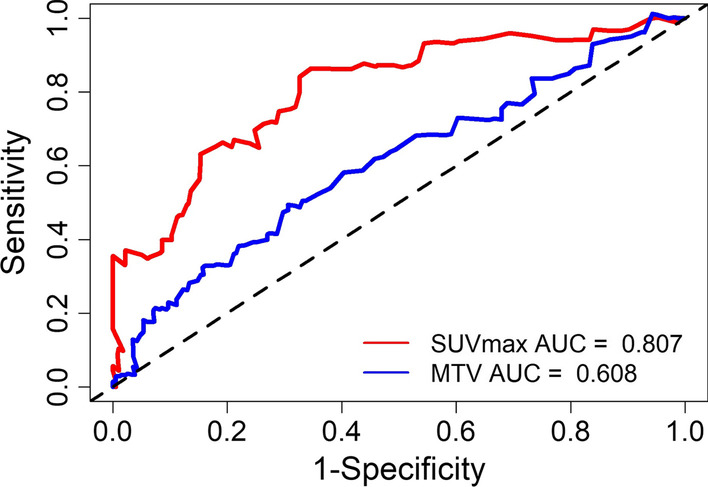
Time-dependent receiver operating characteristic curves (ROC) of maximal standardized uptake value (SUVmax) and metabolic tumor volume (MTV) for survival prediction

**Fig. 3:**
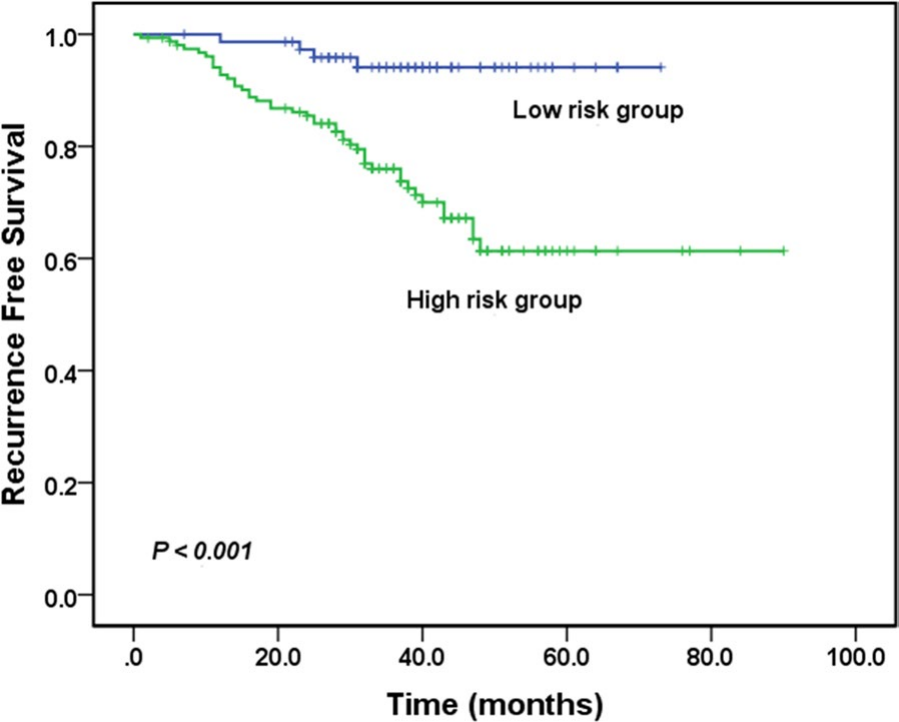
Kaplan–Meier analysis between the high- and low-risk groups. The high-risk group for recurrence was defined as those with IASLC grade 3, the presence of cavity sign, SUV > 3.9, or MTV > 5.4 cm^3^. The 5-year RFS rates were 94.1% and 61.3% for the low- and high-risk groups, respectively

**Fig. 4:**
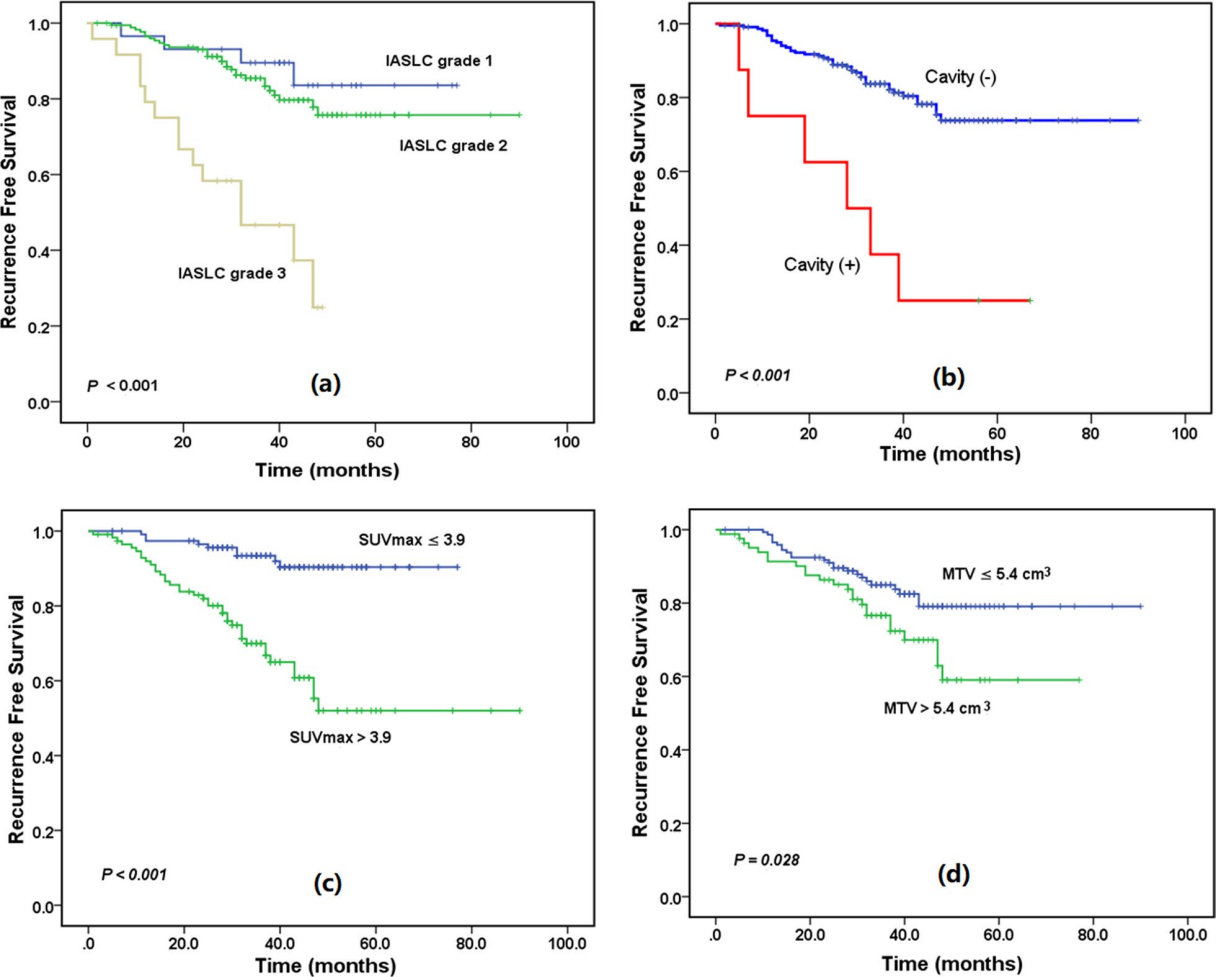
Recurrence-free survival (RFS) based on the **a** International Association for the Study of Lung Cancer Pathology Committee (IASLC) grade, **b** cavity sign, **c** maximal standardized uptake value (SUVmax), and **d** metabolic tumor volume (MTV)

Figure 2 illustrates the time-dependent ROC curves of SUVmax and MTV for survival prediction. Areas under ROC curves were 0.807 and 0.608 for SUVmax and MTV, respectively. The optimal cutoff values were determined to be 3.9 for SUVmax and 5.4 cm^3^ for MTV based on ROC analysis, which implied that patients with SUVmax > 3.9 or MTV > 5.4 cm^3^ were at high risk for recurrence. We defined the high-risk group for recurrence as those with IASLC grade 3, the presence of cavity sign, SUVmax > 3.9, or MTV > 5.4 cm^3^. Figure 3 shows the Kaplan–Meier analysis between the high- and low-risk groups. The RFS significantly differed between the low- and high-risk groups (*P* < 0.001), and the 5-year RFS rates were 94.1% and 61.3% for the low- and high-risk groups, respectively. In the subgroup analyses, RFS rates were all significantly different between the groups of IASLC grade = 3 and IASLC grade < 3 (Fig. 4a), with and without cavity sign (Fig. 4b), SUVmax > 3.9 and SUVmax ≤ 3.9 (Fig. 4c), and MTV > 5.4 cm^3^ and MTV ≤ 5.4 cm^3^ (Fig. 4d).

## Discussion

This study found that SUVmax, MTV, IASLC grade, and cavity sign were independent prognostic factors of recurrence for patients with stage I invasive lung adenocarcinoma manifesting as solid nodules. Based on these independent prognostic factors, we identified patients at high risk of recurrence and found that the 5-year RFS rates were 94.1% for the low-risk group and 61.3% for the high-risk group.

Stage I lung adenocarcinoma is a heterogeneous group, and patients with stage I adenocarcinoma still have the risk of recurrence, especially for solid nodules with invasive adenocarcinoma, which have not been well documented. PET/CT is recommended for lung cancer screening for solid nodules greater than 8 mm in the new lung screening guidance [8]. Tumor metabolic parameters have been used to predict the recurrence risk in early-stage NSCLC. Different cutoff values of SUVmax have been proposed, such as 2.73 [18], 5.5 [19], 5.2 [20], and 4.93 [11]. This difference mainly contributes to the definitions of early-stage NSCLC and pathologic types. We focused on the solid nodules in pathologic stage I invasive adenocarcinoma, a subtype of NSCLC at high risk of recurrence, which has rarely been reported. The methods to determine the optimal cutoff values of SUVmax may also significantly influence the cutoff values of SUVmax. Previous studies [11, 18–20] used the median value or conventional ROC methods to determine the optimal cutoff values of SUVmax. It is well known that lung cancer outcome is time dependent; therefore, ROC curves varying as a function of time may be more appropriate to determine the cutoff values of continuous variables compared with the conventional ROC method [17]. In this study, we applied the time-dependent ROC method to calculate the optimal cutoff value of metabolic parameters and found cutoff values of 3.9 for SUVmax and 5.4 cm^3^ for MTV. In addition, different tumor delineation methods have been used to calculate MTV, including 50%SUVmax, SUV2.5, 40%SUVmax, 42%SUVmax, and mediastinal background SUVmean plus two standard deviations. We adopted the 40%SUVmax method to calculate MTV, which has shown to be effective in our previous studies [21, 22] and other studies [23, 24].

The IASLC proposed a new grading system for invasive lung adenocarcinoma based on the histologic criteria associated with prognosis [15]. Kagimoto et al. [25] then validated the new IASLC grading system. They found that RFS was well stratified by the new grading system among patients with pathologic stage 0 or I but not among patients with stage II or III. We confirmed the significance of the IASLC grading system in prognostic stratification of stage I invasive adenocarcinoma manifesting as solid nodules. We also found that RFS was statistically different between the groups of IASLC grade = 3 and IASLC grade < 3.

We investigated the association between six CT imaging signs and prognosis and found that only the cavity sign was a significant and independent predictor of RFS. Patients with cavity sign were at high risk of recurrence. Tomizawa et al. [12] found that cavity sign was an independent factor for poor prognosis in patients with stage I–IIA primary lung cancer. Zhou et al. [26] reported that the thick-wall cavity could predict worse progression-free survival in lung adenocarcinoma treated with first-line EGFR-TKIs. Chen et al. [27] found cavity formation was a prognostic indicator for pathologic stage I invasive lung adenocarcinoma of ≥ 3 cm in size. Watanabe et al. [28] observed that the overall survival and RFS were significantly shorter in patients with cavitary adenocarcinoma than noncavitary adenocarcinoma, and the cavity formation was an independent prognostic factor in adenocarcinoma. Therefore, the cavity sign may be associated with a poor prognosis for lung cancer. The possible mechanism of the tumor cavity is due to tumor ischemia, infection, and necrosis. Because of the rapid growth of tumor cells, lung tumors could not receive sufficient blood supply, resulting in necrosis of tumor cells, and then tumor cavitation might eventually appear [29]. A larger tumor was more likely to be ischemia, leading to the occurrence of tumor cavity. In addition, previous studies [12, 30] reported that the SUVmax value on FDG PET for lung cancer with tumor cavity was significantly higher than those without tumor cavity. Lung cancers with tumor cavity might have a higher risk of malignancies than those without. Given the complexity of the disease and the challenges, the European Cancer Organization Essential Requirements for Quality Cancer Care (ERQCC) expert group [31] proposed the essential requirements for establishing a high-quality lung cancer service. Although the essential requirements could not be applied to all countries, it was urged that access to multidisciplinary teams and specialized treatment was guaranteed to all patients with lung cancer. Our study may aid in optimizing therapeutic strategies during multidisciplinary discussions and improving survival benefits for those patients.

This study had several limitations. First, this was a retrospective and single-institutional study; further external validation of this study in a large independent population was required. Second, some patients might lose follow-up because of the inherent nature of retrospective studies. Third, PET/CT examination was recommended for solid lung nodules ≥ 8 mm on initial screening [8]; however, many hospitals might not be equipped with PET/CT. Therefore, our recurrence risk evaluation criteria could apply to the patients from the hospitals without PET/CT. Finally, the patient population included was early-stage solid nodules ranging from T1 to T2a, and this cohort was heterogeneous.

In conclusion, we successfully stratified the recurrence risk for patients with stage I invasive lung adenocarcinoma manifesting as solid nodules based on ^18^F-FDG PET/CT, CT imaging signs, and clinicopathological parameters for the first time. Patients with new IASLC grade 3, the presence of cavity sign, SUVmax > 3.9, or MTV > 5.4 cm^3^ were at high risk of recurrence. The 5-year RFS rate in the high-risk group was significantly lower than in the low-risk group (61.3% vs. 94.1%). Our study may help clinicians improve survival benefits and optimize therapeutic strategies for those patients.

## Data Availability

The datasets used and/or analyzed during the current study are available from the corresponding author on reasonable request.

## Abbreviations

CI: Confidence interval
HR: Hazard ratio
MTV: Metabolic tumor volume
NSCLC: Non-small cell lung cancer
RFS: Recurrence-free survival
ROC: Receiver operating characteristic curve
SUVmax: Maximum standardized uptake value
SUVmean: Mean standardized uptake value
TLG: Total lesion glycolysis
TNM: Tumor node metastasis
